# Efficacy of Routinely Used Anticoccidials Against *Eimeria tenella* Field Isolates in Chicken: Bangladesh Perspective

**DOI:** 10.1002/vms3.70579

**Published:** 2025-08-25

**Authors:** Bimal Chandra Karmakar, Nusrat Nowrin Shohana, Anita Rani Dey, Anisuzzaman, Sharmin Aqter Rony, Shirin Akter, Mohammad Zahangir Alam

**Affiliations:** ^1^ Department of Parasitology Bangladesh Agricultural University Mymensingh Bangladesh

**Keywords:** anticoccidials, caecal coccidiosis, drug resistance, *Eimeria tenella*, global index

## Abstract

To evaluate anticoccidial drug efficacy against *Eimeria tenella* in chicken, seven different field isolates were experimented with in battery cages with five commonly used anticoccidials as manufacturer doses, like amprolium (1 g/L), maduramicin (5 ppm), sulphaclozine (2 g/L), toltrazuril (25 mg/L) and amprolium + sulphaquinoxaline (1 g/L). One hundred twelve birds of the Ross strain were raised on a rice husk–littered floor for the first 11 days with ad libitum water and anticoccidial‐free feed, facilitating a standard environment. On Day 12, the birds were divided into seven experimental groups with 16 birds each, and the respective anticoccidials were started for Groups I–V and continued up to 7 days post‐infection. Each bird was infected with 7.5 × 10^4^ sporulated oocysts of *E. tenella* field isolates on Day 14. Global index (GI) was calculated by weight gain, feed conversion ratio (FCR), lesion score, oocyst index and mortality, followed by calculation of %GI_NNC_ to determine the drug efficacy. Data obtained on various parameters were analysed using ANOVA, and the mean values were compared using the Duncan multiple range test through SPSS. The findings revealed that toltrazuril is the best among the experimental drugs, whereas amprolium and sulphaquinoxaline combination holds second place in terms of efficacy. However, the resistance of sulphaclozine was evident in all the isolates, whereas maduramicin showed limited efficacy to partial resistance against caecal coccidiosis. The study strongly recommends toltrazuril against chicken coccidiosis followed by amprolium but highly suggests avoiding long‐term use to maintain drug efficacy.

## Graphical abstract

Among five experimental drugs, toltrazuril showed the highest effectiveness, whereas sulphaclozine was found to be resistant among all the isolates against caecal coccidiosis. The outcome recommends toltrazuril against chicken coccidiosis but highly suggests avoiding long‐term use to minimize drug resistance.

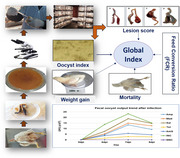

## 1 Introduction

Because of increased human growth, affordability and wide embrace by people of all kinds, poultry production has increased to a large extent. Within 2030, per capita poultry meat and egg consumption is expected to rise by 26% and 41%, respectively (Kawsar et al. 2013). The poultry industry has multidimensional contributions to the livelihood of rural and urban people and plays an economic role in generating income, supplying nutrients and fulfilling food security demands (Birhanu et al. 2023). In the agribusiness sector of Bangladesh, poultry farming is considered one of the dynamic elements that expanded exclusively (2.8% per annum) since the 1990s (Alam et al. 2025). Besides employing 6–8 million people in farming, this sector is greatly contributing to the GDP of Bangladesh (1.85%) (BBS 2023). Despite all these potentialities, different production‐hindering parasitic hidden foes ambush the world poultry sector (Blake et al. 2020) and threaten the global food chain by weakening the potency of the intensive poultry rearing system (Aganovic et al. 2021). Among various parasitic infections, the American Association of Avian Pathologists identified coccidiosis as one of the top diseases of concern affecting broiler and layer farms (Flores et al. 2022).

Avian coccidiosis is caused by multiple species of apicomplexan protozoa, *Eimeria*, which have obligate intracellular properties and cause virulent infectious diseases in the chickens’ intestinal system (Matsubayashi et al. 2020; Lee et al. 2022). Among various *Eimeria* sp., *Eimeria tenella*, the causative agent of caecal coccidiosis, is highly pathogenic (Lee et al. 2022; Sun et al. 2023). The prevalence of coccidiosis around the globe ranges from 7% to 98% (da Silva et al. 2022; Flores et al. 2022), whereas in Bangladesh, the general prevalence is around 10%–42% (Iqbal and Begum 2018). Chicken coccidiosis is strictly host‐specific, and each species occupies a particular predilection site in the intestine (Adem and Ame 2023). *Eimeria* infection destroys host enteric cells, disrupts gut homeostasis, facilitates malabsorption and contributes to the development of subclinical and clinical symptoms of coccidiosis (Yang et al. 2020) and mortality (Wang et al. 2020) and induces vulnerability to necrotic enteritis and zoonotic pathogens like *Salmonella* spp. (Venkatas and Adeleke 2019). Out of all potential poultry diseases, only coccidiosis holds 30% of the overall spending on the pharmacological side (Geng et al. 2021). For the last 25 years, the economic costs induced by chicken coccidiosis skyrocketed from about $0.8 to $14.5 billion per annum (Dalloul and Lillehoj 2006; Blake et al. 2020, 2021). For that reason, it has established itself as a unanimous threat to the poultry industry around the globe (Blake and Tomley 2014).

The disease is endemic in most tropical and subtropical regions where farm management practices mostly include deep litter that creates a suitable environmental inoculum that favours year‐round propagation of *Eimeria* species. The increasing use of intensive farming systems and the associated high stocking densities in farm practices increases the probability of disease persistence (Flores et al. 2022). The cumulative approach of using anticoccidial drugs, providing live anticoccidial vaccines, practising good husbandry and adopting optimum biosecurity is being used as an effective solution to coccidiosis control (Ojimelukwe et al. 2018).

Besides effective biosecurity measures, since 1940, anticoccidial drugs have been safeguarding the poultry industry by prophylactic and therapeutic means (Tian et al. 2017; Ojimelukwe et al. 2018; Blake et al. 2021). Anticoccidials optimize poultry production by benefiting social, economic and environmental aspects of sustainability (Kadykalo et al. 2018). Overall, three categories of anticoccidial drugs are mainly used for coccidiosis control, including synthetic drugs (quinolones, pyridones, alkaloids and thiamine analogues), ionophores and phytotherapy (Gao et al. 2024). In field practice, sulphaclozine sodium, maduramicin, lasalocid, amprolium and toltrazuril are commonly used in Bangladesh (Lovelu et al. 2016). Following antimicrobial resistance, extensive misuse of anticoccidials has also become resistant (Zidar and Žižek 2012; Nilsson et al. 2012), which demands increased veterinary oversight (Attree et al. 2021; Lan et al. 2017).

Studies from various countries, including Nigeria, India and Pakistan, showed different levels of drug resistance and sensitivity against various *Eimeria* species, indicating that the problem is longstanding (Abbas et al. 2008; Ojimelukwe et al. 2018). Due to public and legislative pressure, several anticoccidial drugs have been banned in the European Union (Martins et al. 2022), and demand for ‘drug‐free’ products is increasing in multiple countries (Attree et al. 2021). Even before this demand, researchers sought effective and alternative solutions to anticoccidials, but results showed increased negative impacts on chicken health and performance, including higher litter moisture, burnt feet, necrotic enteritis and airsacculitis (Gaucher et al. 2015; Salois et al. 2017). Additionally, introducing various alternative nutraceuticals like probiotics and vaccines in the diet raised rearing and feed costs.

Given this scenario globally, we have no choice but to find effective anticoccidials, although there might be varying levels of resistance to anticoccidial drugs in Bangladesh's poultry industry. Furthermore, it is concerning that, despite no reports on sulphonamide resistance (Siddiki et al. 2008), there are no recent studies on this issue. Even though it is highly important for human and poultry health, the effectiveness of other commonly used anticoccidials in Bangladesh has been little explored. Therefore, the study aimed to assess the efficacy of commonly used anticoccidials in Bangladesh to recommend the most effective poultry treatments against coccidiosis.

## 2 Materials and Methods

### 2.1 Isolation and Storage of Oocysts From Field Samples

Seven different isolates of *E. tenella* were collected from poultry farms in five key poultry‐concentrated zones of Bangladesh. *E. tenella* field isolates were confirmed by microscopy followed by PCR assay applying species‐specific primers targeting the *ITS‐1* gene (Alam et al. 2022). Isolate 1 was obtained from Mymensingh, Isolates 2 and 3 from Cumilla, Isolate 4 from Tangail, Isolates 5 and 6 from Gazipur and Isolate 7 from Joypurhat. The caeca of suspected dead birds displaying bloody faeces were collected through necropsy. The collected caeca were opened, and discarded faecal materials were removed. Then, caecal mucosal scrapings were obtained using two glass slides. The scrapings were homogenized, and oocysts were isolated through flotation and centrifugation techniques (Qi et al. 2020). Following the method described by Ojimelukwe et al. (2018), the collected oocysts were sporulated in 2.5% potassium dichromate solution at room temperature for 7 days with sufficient aeration by stirring and stored at 4°C until further use.

### 2.2 Preparation of Mass Culture

Day‐old chicks (DOCs) were reared in cage system to generate a mass oocyst culture. At the age of 10 days, following the enumeration through the McMaster technique (Haug et al. 2008), the crop of each bird was directly inoculated with 10^3^ sporulated oocysts (Shirley 1995). At 6–9 days post‐infection (dpi), the presence of oocysts in faecal samples was confirmed through direct microscopy, followed by isolation using floatation and centrifugation techniques (Qi et al. 2020). The isolated oocysts were then temporarily preserved in 2.5% potassium dichromate solution at room temperature for 7 days for sporulation and then stored at 4°C (Ojimelukwe et al. 2018) until further study. Before birds’ inoculation, diluted oocyst samples were repeatedly centrifuged, followed by resuspension in water to remove potassium dichromate (Alam et al. 2022).

### 2.3 Experimental Birds and Their Management

Seven experimental trials were performed on seven different isolates (one for a single isolate). To perform each experiment, 112 DOCs of Ross strain broilers were purchased from Kazi Farms Ltd., Bangladesh. During the first 11 days of age, the birds were reared on the rice husk–littered floor. On Day 12, the birds were transferred to the battery cages by randomly dividing them into seven groups and reared until Day 21. Standard brooding temperature, light and ventilation were maintained, and ad libitum feed (without anticoccidials) and water were supplied. During the first 7 days of age, 90–95°F temperature was maintained, then 5°F temperature was reduced every week until the birds were sacrificed. The cages, utensils, equipment and floor were disinfected twice with a 10% ammonium hydroxide solution spray. Strict biosecurity was maintained before birds’ arrival and during the experimental period, especially regarding visitors’ entry and sanitary practices.

### 2.4 Experimental Design for the Determination of Drug Resistance

For each of the seven isolates (Isolate 1 from Mymensingh, Isolates 2 and 3 from Cumilla, Isolate 4 from Tangail, Isolates 5 and 6 from Gazipur and Isolate 7 from Joypurhat), 112 birds of 12 days old were randomly allocated into seven treatment groups. Each group was subdivided into four replicates with four birds each. From 12 days onwards, birds of Groups I–V were treated with selected anticoccidials up to 7 dpi (Table 1). On Day 14, birds of groups (I–VI) were infected with newly isolated, sporulated 7.5 × 10^4^ oocysts of *E. tenella* (Abbas et al. 2008). Each group of birds’ average weight was measured and recorded before infection on Day 14 and again on 7 dpi (on Day 21). For examination of oocyst index and lesion scoring, birds were sacrificed on Day 21.

**TABLE 1:** Anticoccidial drugs used, manufacturer/supplier name, doses and route of administration.

Treatment groups	Active ingredients	Product name	Manufacturer/Supplier	Dose	Added in
I	Amprolium hydrochloride	Amprol Vet	Eskayef Pharmaceuticals Ltd., Bangladesh	1 g/L	Water
II	Maduramicin ammonium	Coximon	Sunways Bio‐Science Ltd., Europe	5 ppm	Feed
III	Sulphaclozine sodium monohydrate	Coxicure 30%	Renata Pharmaceuticals Ltd., Bangladesh	2 g/L	Water
IV	Toltrazuril	Coxitril Vet	SQUARE Pharmaceuticals Ltd., Bangladesh	25 mg/L	Water
V	Amprolium hydrochloride + sulphaquinoxaline sodium	Cocci‐Off Vet	ACME Laboratories Ltd., Bangladesh	1 g/L	Water
VI	Infected non‐medicated control (INC)	—	—	—	—
VII	Non‐infected non‐medicated control (NNC)	—	—	—	—

*Note*: Group I = amprolium‐medicated group; Group II = maduramicin‐medicated group; Group III = sulphaclozine‐medicated group; Group IV = toltrazuril‐medicated group; Group V = amprolium + sulphaquinoxaline‐medicated group; Group VI = infected non‐medicated control group; Group VII = non‐infected non‐medicated control group.

### 2.5 Treatment Schedule

Five commonly used anticoccidials were used for each of the experiments. Among the seven different groups, birds of Group I were medicated with amprolium in water @ 1 g/L, Group II was medicated with maduramicin @ 5 ppm, mixed in feed, Group III was medicated with sulphaclozine @ 2 g/L, Group IV was medicated with toltrazuril @ 25 mg/L and Group V was medicated with a combination of amprolium and sulphaquinoxaline @ 1 g/L drinking water (Table 1). No drugs were used in Groups VI and VII.

### 2.6 Evaluation of Parameters for Global Index (GI) Calculation

#### 2.6.1 Weight Gain (WG) Calculation

Each of the seven groups’ birds was weighed on Day 14 before giving a challenge infection and again on Day 21 before sacrifice. The body WG of individual birds was calculated by subtracting the weight on Day 14 from the weight on Day 21, before sacrifice.

#### 2.6.2 Feed Conversion Ratio (FCR) Calculation

Data regarding feed intake were recorded from Days 1 to 21 to calculate FCR. It was computed by the ratio of feed consumed by birds and the body WG at Day 21.

#### 2.6.3 Lesion Score Calculation

**FIGURE 1:**
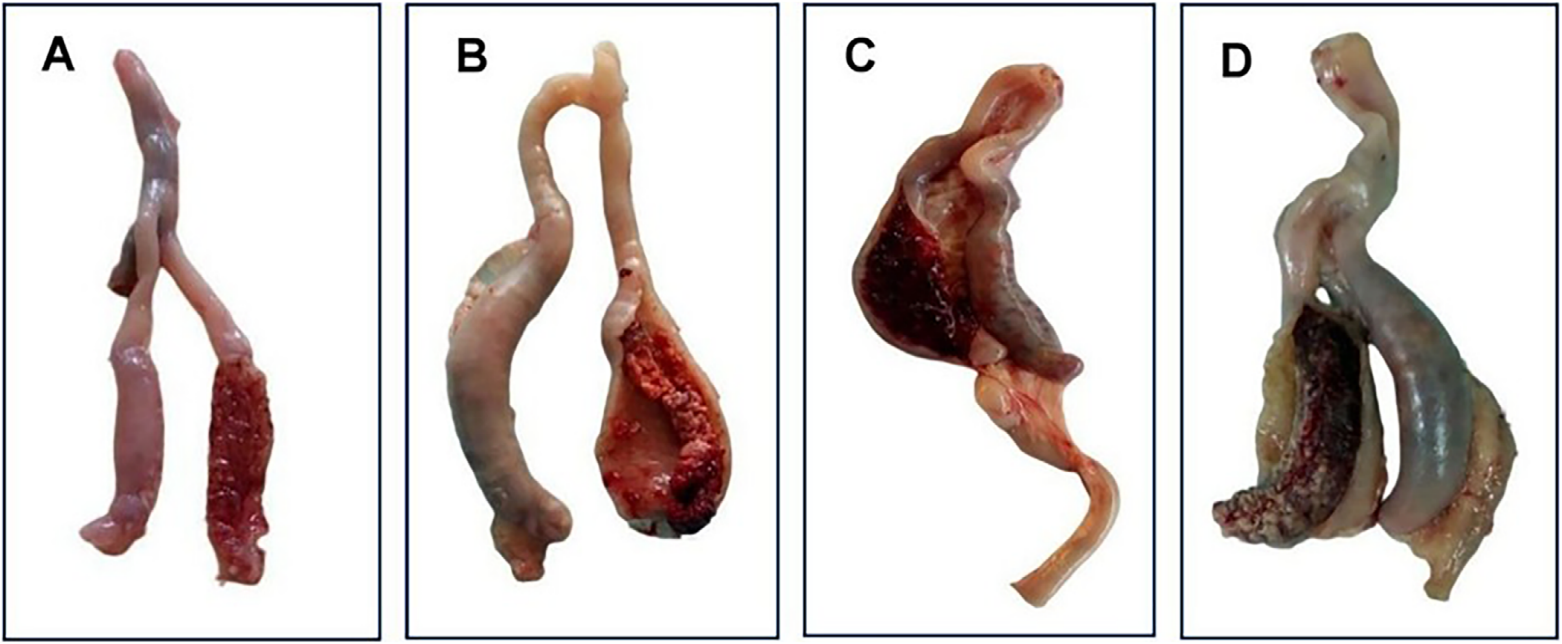
Lesion scoring of caeca. (A) Score 1 having scattered petechiae on the caecal wall without thickening, and caecal contents remain normal; (B) Score 2 with noticeable blood in the caecal contents and slightly thickened caecal wall; (C) Score 3 having large amount of blood or caecal core in caecum with greatly thickened caecal walls; (D) Score 4 with greatly distended caecal wall with blood or large caseous cores with or without faecal debris.

Lesion score was described by the lesions produced following the infection of sporulated oocysts at 7 dpi (Abbas et al. 2008; Ojimelukwe et al. 2018; Wang et al. 2020). Four birds from each treatment group were sacrificed, and then the lesion scores (0–4) were categorized on the basis of the criteria described in Figure 1.

#### 2.6.4 Oocyst Index Calculation

An oocyst index (0–5) was determined by microscopic examination of caecal scrapings of birds sacrificed for lesion scoring at 7 dpi. Oocyst indices were calculated by the method described by Abbas et al. (2008) and Arabkhazaeli et al. (2013). According to this method, no oocyst in a focus indicates 0, 1–10 oocysts per focus indicates 1, 11–20 oocysts per focus scores 2, 21–50 oocysts per focus indicates 3, 51–100 oocysts per focus indicates 4 and more than 100 oocysts per focus indicates 5.

### 2.7 Calculation of GI and Determination of Drug Efficacy

GI was calculated on the basis of the method provided by Stephen et al. (1997). According to the method, it can be expressed as GI **= **%WG_NNC_ − [(*F*_M_ − *F*_NNC_) × 10] − (OI_M_ − OI_INC_) − [(LS_M_ − LS_INC_) − (LS_M_ − LS_INC_) × 2] − (%Motality/2), where GI is global index, WG is weight gain, *F* is feed conversion ratio, OI is oocyst index, LS is lesion score, M is medicated group, INC is infected non‐medicated control group and NNC is non‐infected non‐medicated control group. The GI for each test group was calculated as a percentage of the GI for the NNC. The following five categories were used for testing resistance to anticoccidials: (1) very good efficacy, ≥90% GI_NNC_; (2) good efficacy, 80%–89% GI_NNC_; (3) limited efficacy, 70%–79% GI_NNC_; (4) partially resistant, 50%–69% GI_NNC_; and (5) resistant, <50% GI_NNC_.

### 2.8 OPG Output Calculation to Determine Drug Efficacy

**FIGURE 2:**
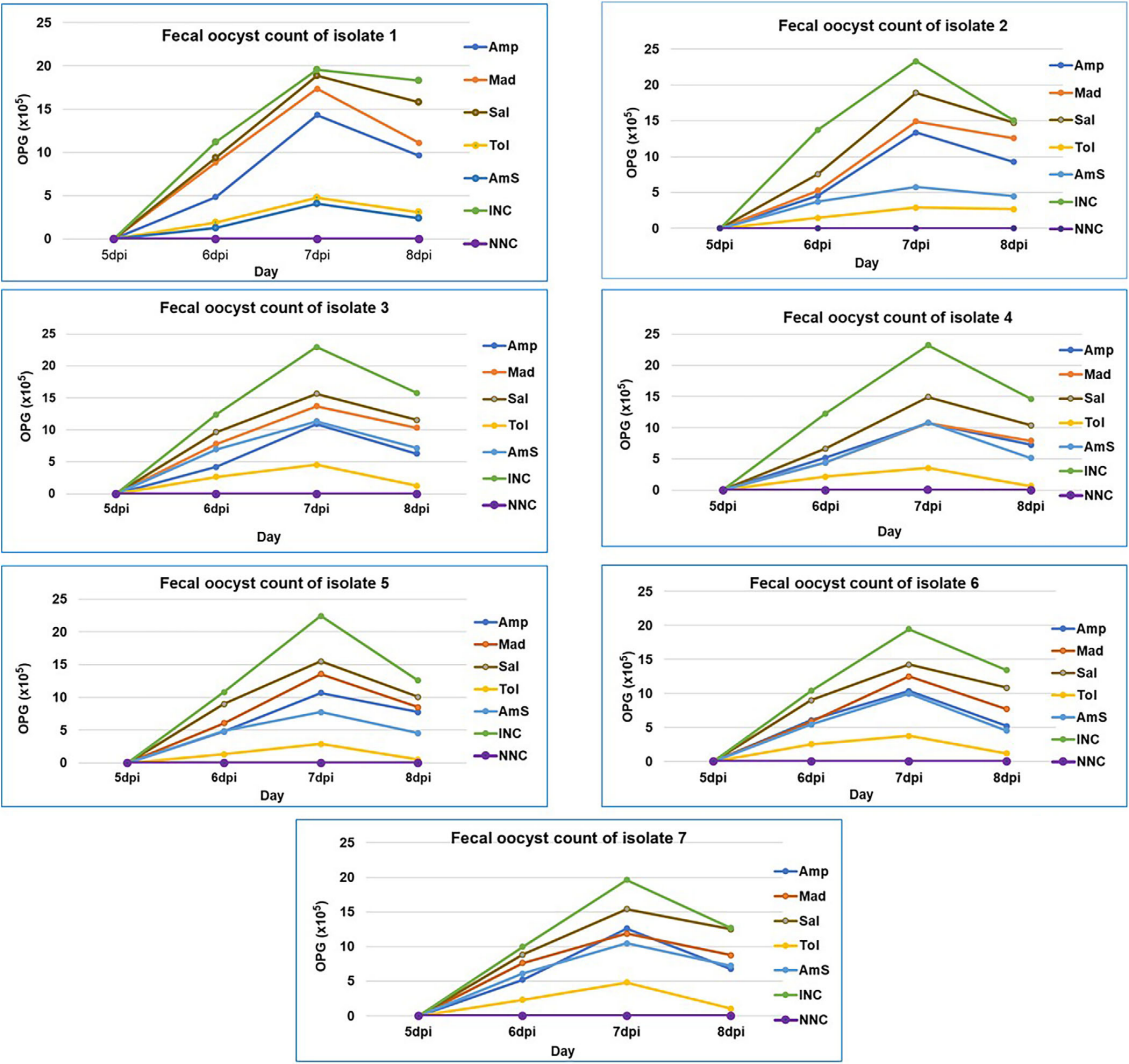
Oocyst shedding in the faeces of different isolates. Amp, amprolium‐medicated group; Mad, maduramicin‐medicated group; Sal, sulphaclozine‐medicated group; Tol, toltrazuril‐medicated group; AmS, amprolium + sulphaquinoxaline‐medicated group; INC, infected non‐medicated control group; NNC, non‐infected non‐medicated control group; OPG, oocyst per gram of faeces; dpi, days post‐infection.

Total faeces from each group were collected from fifth to eighth dpi. For confirming first faecal oocyst excretion, litter samples from each group were checked under a microscope every 12 h. Following collection, each litter sample group was weighed and homogenized separately. The number of oocysts per gram of faeces was enumerated through the McMaster technique (Haug et al. 2008; Alam et al. 2020; Wang et al. 2020) (Figure 2).

### 2.9 Statistical Analysis

Data obtained on various parameters were analysed by IBM SPSS Statistics 22 software. The body WG, FCR, mortality, lesion scores and oocyst index were analysed by one‐way ANOVA and a post hoc analysis using Duncan's multiple range test to identify statistically significant variations. The means of each of the parameters for each isolate were compared separately. The results were recorded as mean, and the differences among group means were considered significant at *p* < 0.05.

## 3 Results

Table 2 summarizes parameters like WG, FCR, mortality, lesion score and oocyst index that were used to calculate the GI and evaluate the performance of different experimental groups in response to the anticoccidials used.

**TABLE 2:** Data regarding various parameters for calculating the global indices of different anticoccidials used against *Eimeria tenella* field isolates.

Isolates	Drugs	WG (g)	FCR (g/g)	Mort (%)	LS	OI
Isolate 1 Mymensingh	Amprolium	108.44^abc^	1.52^ab^	6.25^a^	2.25^b^	2.5^b^
	Maduramycin	99.69^abc^	1.53^ab^	18.75^ab^	3.5^c^	3.5^c^
	Sulphaclozine	96.25^ab^	1.64^bc^	18.75^ab^	3.75^c^	4.0^c^
	Toltrazuril	137.19^cd^	1.43^a^	6.25^a^	2.25^b^	2.25^b^
	Amprolium + sulphaquinoxaline	134.38^bcd^	1.42^a^	6.25^a^	1.25^a^	0.75^a^
	INC	82.19^a^	1.72^c^	31.25^b^	3.75^c^	4.0^c^
	NNC	150.31^d^	1.34^a^	—	—	—
Isolate 2 Cumilla	Amprolium	248.00^b^	1.22^ab^	6.25^a^	2.00^a^	2.00^bc^
	Maduramycin	174.63^ab^	1.38^ab^	18.75^a^	2.25^ab^	3.00^cd^
	Sulphaclozine	186.00^ab^	1.37^ab^	18.75^a^	3.00^bc^	3.75^d^
	Toltrazuril	268.63^b^	1.23^ab^	6.25^a^	1.75^a^	1.50^b^
	Amprolium + sulphaquinoxaline	268.69^b^	1.23^ab^	6.25^a^	1.50^a^	1.75^b^
	INC	120.88^a^	1.54^b^	18.75^a^	3.75^c^	4.00^d^
	NNC	284.44^b^	1.19^a^	—	—	—
Isolate 3 Cumilla	Amprolium	175.06^bc^	1.48^a^	1.56^a^	1.5^a^	2^a^
	Maduramycin	172.5^bc^	1.45^a^	—	2.5^ab^	3.5^ab^
	Sulphaclozine	151.19^b^	1.52^a^	12.5^ab^	2.5^ab^	3.5^ab^
	Toltrazuril	193.94^bc^	1.42^a^	—	1.5^a^	1.75^a^
	Amprolium + sulphaquinoxaline	179.38^bc^	1.48^a^	—	2^a^	2.75^ab^
	INC	59.38^a^	1.93^b^	25^b^	3.25^b^	4^b^
	NNC	208.13^c^	1.38^a^	—	—	—
Isolate 4 Tangail	Amprolium	192.19^cd^	1.31^a^	—	2^ab^	2.75^ab^
	Maduramycin	152^ab^	1.43^bc^	12.5^a^	2.5^bc^	2.5^a^
	Sulphaclozine	140^b^	1.47^c^	12.5^a^	2.75^bc^	3.75^cd^
	Toltrazuril	198.43^d^	1.36^abc^	—	1.5^a^	3^ab^
	Amprolium + sulphaquinoxaline	189.81^cd^	1.37^abc^	—	2.5^bc^	3.25^bc^
	INC	87.31^a^	1.73^d^	25^b^	3.25^c^	4.25^d^
	NNC	217.5^d^	1.28^a^	—	—	—
Isolate 5 Gazipur	Amprolium	294.06^b^	1.39^a^	—	1.75^ab^	2.25^ab^
	Maduramycin	218.13^ab^	1.57^ab^	3.13^a^	2.5^bc^	2.75^bc^
	Sulphaclozine	209.06^ab^	1.58^ab^	—	2.75c	3.25^cd^
	Toltrazuril	300.63^bc^	1.42^a^	—	1.75^ab^	1.5^a^
	Amprolium + sulphaquinoxaline	261.56^bc^	1.3^a^	—	1.5^a^	1.5^a^
	INC	137.81^a^	1.8^b^	25^b^	3.5^d^	4^d^
	NNC	348.44^c^	1.3^a^	—	—	—
Isolate 6 Gazipur	Amprolium	268.75^bcd^	1.42^ab^	—	2.00^ab^	2.00^a^
	Maduramycin	235.63^bc^	1.51^ab^	—	2.50^b^	2.25^ab^
	Sulphaclozine	210.94^ab^	1.56^bc^	—	2.75^bc^	3.25^bc^
	Toltrazuril	303.75^cd^	1.38^ab^	—	1.50^a^	1.75^a^
	Amprolium + sulphaquinoxaline	246.56^bc^	1.34^a^	—	1.50^a^	2.00^a^
	INC	163.75^a^	1.72^c^	—	3.50^c^	4.00^c^
	NNC	321.88^d^	1.33^a^	—	—	—
Isolate 7 Joypurhat	Amprolium	251.69^b^	1.18^a^	6.25^a^	2^ab^	2^ab^
	Maduramycin	218.94^b^	1.27^a^	6.25^a^	2.5^b^	2.25^ab^
	Sulphaclozine	209.44^b^	1.31^a^	6.25^a^	2.75^bc^	3^bc^
	Toltrazuril	267.88^b^	1.17^a^	—	1.5^a^	1.75^a^
	Amprolium + sulphaquinoxaline	228.44^b^	1.17^a^	6.25^a^	1.5^a^	2^ab^
	INC	100^a^	1.62^b^	37.5^b^	3.5^c^	4^c^
	NNC	282.13^b^	1.15^a^	—	—	—

*Note*: “a–d” means sharing the same superscripts within each section do not differ (*p* ≤ 0.05).

Abbreviations: AWG, average weight gain; FCR, feed conversion ratio; INC, infected non‐medicated control; LS, lesion score; NNC, non‐infected non‐medicated control; OI, oocyst index; WG, weight gain.

From Table 2, it is evident that in all the cases, the WG of NNC group was higher than other medicated groups, whereas the INC group showed the lowest WG. In most of the cases, there was no significant difference observed between the WGs of birds treated with toltrazuril and birds treated with amprolium + sulphaquinoxaline (*p* > 0.05). No significant differences of WG were recorded among the birds of different medicated and NNC groups in Isolate 7. However, the WG of different medicated and NNC groups varied significantly with INC group (*p* < 0.05).

FCR values of the positive control (INC) group in all the isolates exhibited the poorest result and differed significantly (*p* < 0.05) from other medicated groups and NNC. However, in all the isolates, the FCR of NNC groups revealed the best output. Notably, birds treated with toltrazuril and amprolium + sulphaquinoxaline groups displayed very close FCR, and no significant differences were observed (*p* < 0.05).

In the case of all the isolates, INC groups revealed significantly higher mortality percentages compared to the medicated groups (*p* < 0.05), whereas no mortality was recorded in NNC groups. Among the medicated groups of Isolates 1 and 2, sulphaclozine‐ and maduramicin‐treated groups showed the highest mortality (18.75%) and significantly varied from the others. Similar trends were also observed (12.5%) in Isolate 4. In the case of Isolates 3–7, no mortality was observed in the toltrazuril‐treated groups. Likewise, amprolium + sulphaquinoxaline‐medicated groups showed no mortality in Isolates 3–5 and 7.

The highest lesion scores were observed in INC group in all the isolates, and in most cases, no significant differences were observed with sulphaclozine‐ and maduramicin‐medicated groups (*p* < 0.05). Among different medicated groups, sulphaclozine‐treated groups showed higher lesion scores in all isolates, depicting a significant difference (*p* < 0.05) with other medicated groups. No lesion scores were recorded in NNC groups in all the cases.

The oocyst index value of INC groups showed higher results in all the isolates, and in most cases, no significant differences were observed with sulphaclozine‐medicated groups (*p* < 0.05). However, no oocyst was found in the NNC group. In most of the isolates, except Isolate 1, among the medicated groups, the lowest oocyst indices were recorded in toltrazuril‐medicated groups (*p* < 0.05) (Table 2).

Applying data from Table 2, global indices were calculated to evaluate the efficacy of selected anticoccidials against seven isolates. The GI was computed as the percentage of the corresponding GI for the NNC (%GI_NNC_) and summarized in Table 3. The best result was observed from the toltrazuril‐medicated groups in all the isolates. In Isolate 5, the %GI_NNC_ for toltrazuril was 85.97, indicating good efficacy. However, in Isolates 1–4, 6 and 7, the %GI_NNC_ were 92.24, 90.84, 90.10, 89.54, 90.61 and 92.22, respectively, indicating very good efficacy. The %GI_NNC_ of amprolium‐medicated groups for all seven isolates (1–7) were 81.13, 81.55, 84.40, 82.57, 80.85, 81.95 and 80.31, respectively, exhibiting good efficacy. On the other hand, except for Isolate 2 (which showed very good efficacy with %GI_NNC_ 90.02), the amprolium + sulphaquinoxaline‐medicated group showed %GI_NNC_ 87.83, 85.99, 79.48, 81.40, 82.14 and 80.54, respectively, demonstrating good efficacy. The result of %GI_NNC_ of maduramicin against *E. tenella* field isolates (1–7) was 68.42, 51.23, 76.58, 58.77, 70.09, 68.67 and 73.18, respectively. That means the drug showed limited efficacy against Isolates 3, 5 and 7 and partial resistance against Isolates 1, 2, 4 and 6. The %GI_NNC_ of sulphaclozine‐medicated groups were 33.91, 54.49, 65.23, 52.19, 58.05, 63.26 and 68.40, respectively, against seven different isolates. The results indicated that sulphaclozine showed partial resistance to Isolates 2–7 and resistance to Isolate 1 (Table 3).

**TABLE 3:** Efficacy of anticoccidial drugs against different *Eimeria tenella* field isolates.

Isolates	Drugs used	GI	%GI_NNC_	Efficacy status
Isolate 1 Mymensingh	Amprolium	70.25	81.13	GE
	Maduramycin	59.24	68.42	PR
	Salphaclozine	29.36	33.91	R
	Toltrazuril	79.87	92.24	VGE
	Amprolium + sulphaquinoxaline	76.05	87.83	GE
	INC	30.26	34.95	—
	NNC	86.59	100.00	—
Isolate 2 Cumilla	Amprolium	90.96	81.55	GE
	Maduramycin	33.67	51.23	PR
	Salphaclozine	37.39	54.49	PR
	Toltrazuril	101.25	90.84	VGE
	Amprolium + sulphaquinoxaline	101.15	90.02	VGE
	INC	30.34	27.23	—
	NNC	111.50	100.00	—
Isolate 3 Cumilla	Amprolium	91.47	84.40	GE
	Maduramycin	61.34	76.58	LE
	Sulphaclozine	47.91	65.23	PR
	Toltrazuril	103.57	90.10	VGE
	Amprolium + Sulphaquinoxaline	93.97	85.99	GE
	INC	12.46	11.24	—
	NNC	110.50	100.00	—
Isolate 4 Tangail	Amprolium	91.45	82.57	GE
	Maduramycin	65.20	58.77	PR
	Sulphaclozine	57.76	52.19	PR
	Toltrazuril	99.18	89.54	VGE
	Amprolium + sulphaquinoxaline	88.06	79.48	LE
	INC	24.42	22.05	—
	NNC	110.75	100.00	—
Isolate 5 Gazipur	Amprolium	88.53	80.85	GE
	Maduramycin	43.01	70.09	LE
	Sulphaclozine	39.87	58.05	PR
	Toltrazuril	99.33	85.97	GE
	Amprolium + Sulphaquinoxaline	88.42	81.40	GE
	INC	24.24	25.85	—
	NNC	111.00	100	—
Isolate 6 Gazipur	Amprolium	89.89	81.95	GE
	Maduramycin	53.07	68.67	PR
	Sulphaclozine	45.87	63.26	PR
	Toltrazuril	105.71	90.61	VGE
	Amprolium + sulphaquinoxaline	91.09	82.14	GE
	INC	49.32	45.00	—
	NNC	111.00	100	—
Isolate 7 Joypurhat	Amprolium	89.56	80.31	GE
	Maduramycin	56.66	73.18	LE
	Sulphaclozine	51.88	68.40	PR
	Toltrazuril	102.30	92.22	VGE
	Amprolium + sulphaquinoxaline	89.53	80.54	GE
	INC	13.31	11.59	—
	NNC	111.00	100.00	—

*Note*: GI, global index (calculated from different parameters); %GI_NNC_, %WG_NNC_ − [(FM − *F*_NNC_) × 10] − (OI_M_ − OI_INC_) − [(LS_M_ − LS_INC_) × 2)] − (%mortality/2), where WG is weight gain; *F* is FCR (feed conversion ratio); OI is oocyst index; LS is lesion score; M is medicated group; INC is infected non‐medicated control group; NNC is non‐infected non‐medicated control group; VGE is very good efficacy; GE is good efficacy; LE is limited efficacy; PR is partially resistant; and R is resistant.

To evaluate the OPG output, litter samples were collected from the fifth to eighth dpi. Oocyst shedding starts at sixth dpi, becomes peak at seventh dpi and starts declining at eighth dpi. In all seven isolates, the highest OPG outputs were recorded in INC groups, and the lowest OPG counts were recorded in NNC groups. Among the medicated groups, toltrazuril‐treated groups showed the least OPG count, except for Isolate 1 where amprolium + sulphaquinoxaline‐medicated group shed lowest OPG. On the other hand, sulphaclozine‐medicated groups shed the highest oocysts per gram of faeces (Figure 2).

## 4 Discussion

Among the control strategies of coccidiosis, anticoccidial drug application is one of the most popular and commonly practised techniques. However, the irrational long‐term use coupled with the availability of similar drugs all over a country may influence drug resistance and its consequences (Chapman et al. 2010).

Amprolium, toltrazuril, maduramicin, sulphaquinoxaline, sulphaclozine and sulphadimethoxine are commonly available anticoccidial chemoprophylaxis in Bangladesh (Lovelu et al. 2016; Rony et al. 2021). Reportedly, the susceptibility of coccidian protozoa towards anticoccidials like sulphonamides is decreasing, indicating the emergence of drug resistance (Siddiki et al. 2008). Aside from this drug, the rest of the list was largely unexplored for a long period. Here, the efficacy of amprolium, toltrazuril, maduramicin, sulphaclozine and a combination of amprolium + sulphaquinoxaline was evaluated, comparing GI parameters to scan the current scenario at the field level.

In the 1980s and 1990s, the performance index and anticoccidial index were routinely employed formulas for assessing the sensitivity or resistance of anticoccidial drugs. In these, FCR was not given importance, although feed price occupies a major share of broiler chicken production costs (Jordan and Pattinson 1998). Meanwhile, the newly devised formula of Stephen et al. (1997) encompasses all five parameters (WG, FCR, lesion score, oocyst index and mortality), and many researchers found close correspondence between resistant results and clinical findings (Abbas et al. 2008). Therefore, GI is a proven valid tool for evaluating anticoccidial resistance.

The selection of multiple field strains instead of one or two strengthens the conclusive summary of resistance under field conditions. To have the essence of the real field resistance scenario, seven isolates of *E. tenella* were selected from the poultry farms of five different districts of Bangladesh. Among the isolates, two were collected from Cumilla, one from Mymensingh, one from Tangail, two from Gazipur and one from Joypurhat. In the present study, toltrazuril stood as the most effective anticoccidial and in agreement with several studies (Grief 2000; Dhillon et al. 2004; Lovelu et al. 2016). However, Sun et al. (2023) and Ojimelukwe et al. (2018) did not comply with our finding, as they found slight resistance to toltrazuril against *Eimeria* field isolates in China and Nigeria. In the case of amprolium, it also showed good efficacy and was consistent with Rony et al. (2021) and Lovelu et al. (2016). Meanwhile, this outcome conflicted with Arabkhazaeli et al. (2013), as they reported limited efficacy. Although the findings suggest that the combination of amprolium and sulphaquinoxaline is effective, Ojimelukwe et al. (2018) differed in this case, which may be due to long‐term use or genetic diversity of *Eimeria* parasites. Moreover, the use of sulpha drugs may raise questions about the effectiveness of that combination, as researchers have discouraged their use for a long time (Gill and Bajwa 1979; Krylov and Zaionts 1981; Siddiki et al. 2008). The %GI_NNC_ values of maduramicin of the study isolates revealed resistance to a great extent. Although the findings differed from those of Abbas et al. (2008), who reported varying degrees of sensitivity of maduramicin against chicken coccidiosis, McDougald et al. (1990) found similar findings in Turkey. However, the results of Isolates 3, 5 and 7 agreed with the findings of Yadav and Gupta (2001). Meanwhile, sulphaclozine showed partial to full resistance against some selected chicken anticoccidials that agreed with the findings of Siddiki et al. (2000), as it was commonly used in Bangladesh against chicken coccidiosis since the early seventies (Siddiki et al. 2008). However, Harun‐or‐Rashid et al. (2016) reported sulphaclozine as an effective anticoccidial.

The development of partial resistance of specific field study isolates against different drug types like ionophores (maduramicin) and sulpha drugs (sulphaclozine) may indicate their long‐term and indiscriminate use with suboptimal dosing followed by gradual reduction in the susceptibility to coccidia. Moreover, genetic recombination or mutation may orchestrate the mechanisms of multiple anticoccidial resistance of a single study isolate. In this study, multiple isolates showed very good efficacy against toltrazuril and amprolium, whereas those isolates also revealed partial to full resistance against maduramicin and sulphaclozine. This interpretation may lead to the fact that a single *Eimeria* species consists of multiple strains, where environmental selection pressure and drug handling history may be the vital predisposing factors responsible for these kinds of findings. Besides, if every bird does not receive an equal dose, there is the potential for some birds to acquire wild‐type strains, which may lead to asynchronous development of immunity and ultimately to resistance.

Aside from faulty and ineffective dosing, strain variation based on geographical locations may suggest customized plans for effective coccidiosis control strategies for individual countries, and for that, there are reports of anticoccidials that are resistant to one country but highly sensitive to others.

Faecal oocyst shedding represents the impact of disease, demonstrates parasitic infection (Lee et al. 2009) and is also used to categorize the risk of poultry flocks (Haug et al. 2008). Therefore, the faecal oocyst output was enumerated in all the medicated and control groups from the fifth to the eighth dpi. The highest oocyst shedding was recorded in the INC groups, and no oocysts were found in the NNC groups. These findings are compatible with the results of Ojimelukwe et al. (2018). Among the medicated groups, birds of toltrazuril‐medicated groups excreted the least oocysts, whereas the highest concentration of oocysts was observed in sulphaclozine‐treated groups. The findings are not compatible with Ojimelukwe et al. (2018), who recorded the highest oocyst output in toltrazuril‐medicated group and the lowest concentration in the amprolium‐medicated group. Siddiki et al. (2008) isolated varying quantities of oocysts in a study of multiple doses of sulphaclozine application. We detected the first faecal oocysts at sixth dpi, whereas Ojimelukwe et al. (2018) found oocyst excretion at fifth dpi. However, in both studies the highest OPG output was recorded at seventh dpi. Jordan et al. (2011) also studied oocyst output post‐challenge and found peak faecal oocyst production on 7–9 dpi. It was depicted that the highest oocyst grade exhibits a large OPG count in all the cases.

## 5 Conclusion

Among the drugs used, toltrazuril showed the highest efficacy against the field isolates. On the contrary, sulphaclozine depicted varying degrees of resistance. Amprolium alone or in combination showed good efficacy, whereas maduramicin showed limited efficacy in some isolates and varying degrees of resistance in some cases. So, the findings highly suggest toltrazuril as an effective drug of choice against caecal coccidiosis. It also recommends amprolium alone or in combination with sulphaquinoxaline. However, sulphaclozine and maduramicin should be avoided as anticoccidials for chicken coccidiosis in Bangladesh.

## Author Contributions

**Bimal Chandra Karmakar**: methodology, investigation, software, data curation, formal analysis, writing – review and editing, writing – original draft, visualization. **Nusrat Nowrin Shohana**: methodology, writing – original draft, writing – review and editing, software. **Anita Rani Dey**: writing – review and editing, formal analysis. **Anisuzzaman**: conceptualization, writing – review and editing. **Sharmin Aqter Rony**: supervision, methodology. **Shirin Akter**: methodology, writing – review and editing. **Mohammad Zahangir Alam**: conceptualization, methodology, supervision, funding acquisition, writing – review and editing, project administration, resources, visualization.

## Ethics Statement

The survey was performed following the guidelines approved by the Animal Welfare and Experimentation Ethics Committee of Bangladesh Agricultural University, Mymensingh (Approval number: AWEEC/BAU/2023 (46)).

## Conflicts of Interest

The authors declare no conflicts of interest.

## Peer Review

The peer review history for this article is available at https://www.webofscience.com/api/gateway/wos/peer‐review/10.1002/vms3.70579.

## Data Availability

The data that support the findings of this study are available from the corresponding author on reasonable request.
